# Growth of Multiorientated Polycrystalline MoS_2_ Using Plasma-Enhanced Chemical Vapor Deposition for Efficient Hydrogen Evolution Reactions

**DOI:** 10.3390/nano10081465

**Published:** 2020-07-27

**Authors:** Na Liu, Jeonghun Kim, Jeonghyeon Oh, Quang Trung Nguyen, Bibhuti Bhusan Sahu, Jeong Geon Han, Sunkook Kim

**Affiliations:** 1 School of Advanced Materials Science & Engineering, Sungkyunkwan University (SKKU), Suwon, Gyeonggi-do 16419, Korea; naliufit9@gmail.com (N.L.); jhuni7803@gmail.com (J.K.); raymond.lucid@gmail.com (J.O.); nqtrungdt@gmail.com (Q.T.N.); 2 Department of Electrical Engineering and Computer Science, Graduate School of Engineering, Center for Low-Temperature Plasma Sciences, Nagoya University, Nagoya 464-8601, Japan; bibhutisahu@gmail.com; 3 Thai-Korea Research Collaboration Research Center (TKRCC), Science and Technology Park (STeP), Chiang Mai University, Chiang Mai 50200, Thailand; hanjg5445@gmail.com

**Keywords:** hydrogen evolution reaction, MoS_2_, plasma-enhanced chemical vapor deposition

## Abstract

Molybdenum disulfide (MoS_2_) has attracted considerable attention as a promising electrocatalyst for the hydrogen evolution reaction (HER). However, the catalytic HER performance of MoS_2_ is significantly limited by the few active sites and low electrical conductivity. In this study, the growth of multiorientated polycrystalline MoS_2_ using plasma-enhanced chemical vapor deposition (PECVD) for the HER is achieved. The MoS_2_ is synthesized by sulfurizing a sputtered pillar-shaped Mo film. The relatively low growth temperature during the PECVD process results in multiorientated MoS_2_ with an expanded interlayer spacing of ~0.75 nm, which provides abundant active sites, a reduced Gibbs free energy of H adsorption, and enhanced intralayer conductivity. In HER applications, the PECVD-grown MoS_2_ exhibits an overpotential value of 0.45 V, a Tafel slope of 76 mV dec^−1^, and excellent stability in strong acidic media for 10 h. The high HER performance achieved in this study indicates that two-dimensional MoS_2_ has potential as an electrocatalyst for next-generation energy technologies.

## 1. Introduction

Rapid industrial growth has led to accelerating energy consumption and increased contamination of the environment. Developing clean sources of energy is necessary for reducing the amounts of pollutants and greenhouse gases released by the consumption of fossil fuels, such as coal [1]. Over the past few decades, alternative types of energy that can be converted into electrical energy have been widely studied, such as solar energy, geothermal power, and biomass [2]. H, which has the highest energy density of all chemical fuels, is also a promising ecofriendly energy source [3]. However, the production of H via the steam-reforming method used in industry causes environmental pollution owing to the generation of CO_2_ at high temperatures, similar to the use of fossil fuels [4]. In contrast, the hydrogen evolution reaction (HER) is a sustainable and efficient approach to generate H from water [5].

The HER needs a catalyst to reduce the overpotential and increase efficiency, which is important for the electrochemical process [6]. Acidic electrolytes are suitable for the HER because these units are compact and can potentially run in the reverse mode to produce electricity [7]. The most efficient electrocatalysts for the HER under acidic conditions are based on noble metals, particularly Pt [8]. Pt-based materials have excellent kinetics for driving the HER. Although Pt has a low overpotential and high energy efficiency, it is an expensive and limited resource and thus not suitable for practical use.

In the past few years, MoS_2_-based electrocatalysts have been considered as promising alternatives to Pt because of their low price and large reserves [9]. Additionally, experimental and theoretical studies indicated that MoS_2_ has high activity for electrochemical H evolution [10]. Density functional theory calculations indicated that the Gibbs free energy for H adsorption is close to thermo-neutral, making MoS_2_ a promising HER catalyst. There is a close association between the electronic structure and the catalytic activity of a catalyst [9]. Vertically aligned and amorphous MoS_2_ with many unsaturated S atoms having abundant active sites has yielded significant improvements in the HER, including a higher current density, improved turnover frequency, and reduced overpotential [11,12,13]. Owing to its excellent electrocatalytic properties, MoS_2_ has attracted considerable interest in the field of alternative energy.

Although amorphous MoS_2_ has been considered as an efficient HER catalyst with many active edges, it leads to high solubility and electrochemical instability in an acid electrolyte [14]. Various strategies have been developed to increase the number of active sites and enhance the catalytic performance of MoS_2_. Chemical vapor deposition (CVD) [15,16,17,18], atomic layer deposition (ALD) [19,20], and hydrothermal methods [21,22] have been widely used to synthesize MoS_2_. High temperatures are essential for MoS_2_ synthesis but degrade HER performance [18]. Plasma-assisted synthesis techniques have been shown to reduce the required temperature for MoS_2_ synthesis [23,24,25]. However, the growth of MoS_2_ for HER applications using plasma-enhanced CVD (PECVD) has not been attempted.

In this study, we synthesized multiorientated polycrystalline MoS_2_ films using a two-step process and employed them for the HER. The two-step process has conducted radiofrequency (RF) sputtering and PECVD sulfurization. The MoS_2_ growing along the surface of pillar-shaped Mo films exhibited multiorientated edges and an enlarged interlayer spacing, which were expected to enhance the catalytic HER performance. The synthesized MoS_2_ film exhibited an overpotential of 0.45 V for a current density of 10 mA cm^−2^ and a Tafel slope of 76 mV dec^−1^. Additionally, the MoS_2_ exhibited high stability under strong acidic media for 10 h. The excellent electrocatalytic ability of our MoS_2_ films indicates their great potential for application in clean energy production.

## 2. Materials and Methods

### 2.1. Growth of MoS_2_

**Figure 1:**
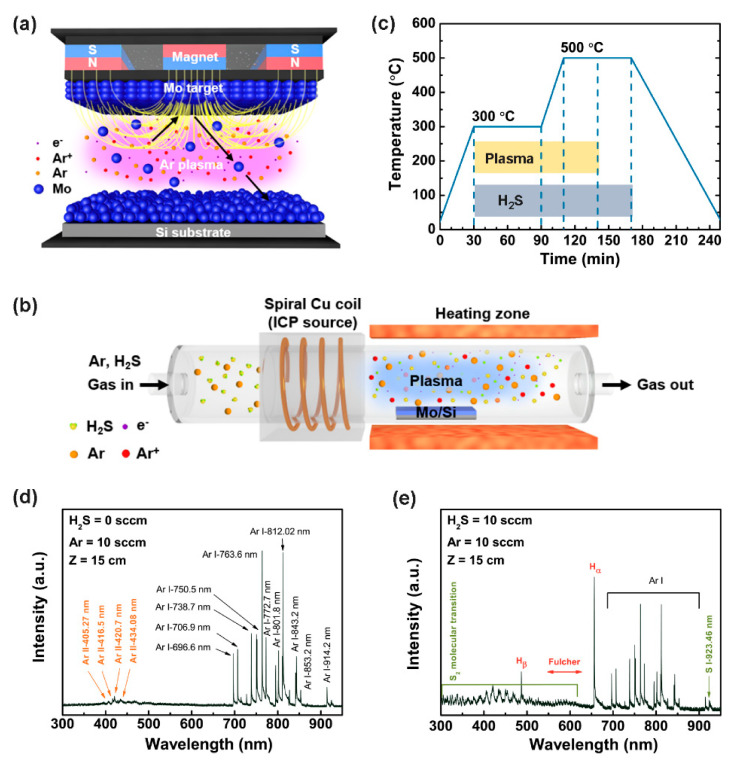
Schematics of the experimental setup for the two-step synthesis of MoS_2_: (**a**) schematic of the radiofrequency (RF) magnetron sputtering system for Mo deposition on a p-type Si substrate; (**b**) schematic of the inductively coupled plasma (ICP)-assisted plasma-enhanced chemical vapor deposition (PECVD) method for sulfurizing the Mo film to obtain MoS_2_. (**c**) Temperature profile with plasma and H_2_S gas flow during the PECVD growth process. (**d**) Typical optical emission spectroscopy (OES) spectrum of pure Ar plasma at a power of 300 W. (**e**) OES spectrum of Ar and H_2_S plasma at a power of 300 W.

MoS_2_ was grown on a monocrystalline p-type Si (resistance of 0.005 Ω) substrate through sputtering and PECVD, as described in Figure 1a,b. Before Mo sputtering on the Si substrate, a cleaning process was performed using an ultrasonicator with acetone and isopropyl alcohol for 15 min each. Then, the Si substrate was immersed in 10% HF for 5 min to remove the native oxide layer. One piece of the Si substrate (1 cm × 2 cm) was placed in the middle of the substrate holder. Presputtering was conducted for 5 min before the Mo deposition. The deposition was performed for 50 min at room temperature, with an RF power of 150 W. The distance between the Mo target and the substrate holder was approximately ~10 cm. Ar gas was injected into the chamber, and the pressure for depositing Mo on the Si substrate was set as 7 mTorr. After the Mo film deposition, pure Ar gas was flowed into the chamber for a few minutes to stabilize the equipment. The detailed information about the sputter system is shown in Figure S1.

After the sputtering was complete, the Mo-deposited Si substrate was removed from the sputtering chamber and placed on a ceramic boat for PECVD growth. It was placed in a furnace, 15 cm away from inductively coupled plasma (ICP). The chamber was heated with 60-sccm Ar gas. When the temperature reached 300 °C, ICP with a power of 300 W was turned on for 1 h, and 20 sccm H_2_S gas was injected for 2 h and 20 min. The PECVD growth occurred at 0.5 Torr, under an H_2_S flow. After 1 h at 300 °C, the chamber was heated from 300 to 500 °C in 20 min. The temperature was maintained for 1 h, and then the chamber was cooled slowly under Ar gas with a flow rate of 60 sccm.

### 2.2. Characterization

The optical emission spectroscopy (OES) spectra under different operating conditions were acquired using an optical fiber and a spectrometer (Acton Spectra Pro 500i, Acton, MA, USA) with a resolution of 0.05 nm. The emission intensities of various excited species in the magnetron sputtering plasmas were investigated in the broad wavelength range of 300–900 nm. The crystallinity and chemical composition of the MoS_2_ film were analyzed using Raman spectroscopy and X-ray photoelectron spectroscopy (XPS) (MXP10, K-Alpha, Thermo Fisher Scientific, Waltham, MA, USA) respectively. The Raman spectra were measured using a multipurpose spectrometer (LabRAM HR800, Horiba Jobin Yvon, Kyoto, Japan) with 514.5-nm laser excitation. The binding energies of the XPS analyst were calibrated with C 1s at 284.5 eV. To obtain crystallization and orientation information about the MoS_2_, X-ray diffraction (XRD) analysis (MXD10, SmartLab, Rigaku, Tokyo, Japan corporation voltage = 12 kV) was performed in the scanning range of 2*θ* = 10° to 90° at a scanning rate of 4° min^−1^, using Cu Kα radiation. The thicknesses and morphologies of Mo and MoS_2_ were determined using atomic force microscopy (AFM) (XE7, Park Systems, Suwon, South Korea) with the contact-mode operation. A contact cantilever (EFM Cantilever, Park Systems, Suwon, South Korea) was used to obtain 10 × 10-µm^2^ topographic images, and the scan rate was 0.1 Hz. The morphology and elemental composition of the surface of the MoS_2_ film were characterized using scanning electron microscopy (SEM) (JSM 7500F, JEOL, Tokyo, Japan) together with energy-dispersive X-ray spectroscopy (EDS; JSM 7500F, JEOL and JEM ARM 200F, JEOL, Tokyo, Japan) and high-resolution transmission electron microscopy (HRTEM) (JEM ARM 200F at 80 kV, JEOL, Tokyo, Japan). The HRTEM imaging sample was fabricated using a focused ion beam (NX2000, Hitachi, Tokyo, Japan).

Electrochemical measurements were conducted to evaluate the HER performance of each synthesized catalyst in a strongly acidic condition. Each catalyst was employed as a working electrode, and its exposed area was fixed as 1 cm × 1 cm. A Pt plate was used as a counter electrode, and Ag/AgCl with a 3 M NaCl internal solution (ALS, Japan, Tokyo, Japan) was used as a reference electrode. The electrochemical tests were conducted at room temperature in a 0.5 M H_2_SO_4_ (pH ≈ 0) solution using an Ivium-n-Stat (Ivium Technology, Eindhoven, Netherlands). The counter and working electrodes were separated by a proton-exchange membrane to prevent Pt transfer from the anode to the cathode. All the potentials reported in this work were corrected with respect to a reversible hydrogen electrode (RHE) by adding a value of (0.209 + 0.059 × pH) V. The electrocatalytic activities for the HER were determined via linear sweep voltammetry with a scan rate of 5 mV s^−1^. Si, Mo on Si, and Pt (20 nm) on Si were tested under the same conditions, for comparison. The polarization curves were converted into a semi-log plot, and the linear region of each result was selected to extract the Tafel slope values (with the unit of mV dec^−1^). Electrochemical impedance spectroscopy (EIS) was performed to determine the interfacial characteristics of the as-synthesized catalysts in the frequency range of 100 kHz to 100 mHz and measure the excitation signal of 10 mV (root-mean-square (RMS)) at an open-circuit potential (OCP). To evaluate the electrochemically active surface area (ECSA), cyclic voltammetry (CV) was performed in the potential range of 0.10–0.20 V vs. RHE, where the non-Faradaic process occurred. The double-layer capacitance (*C_dl_*) was determined by plotting the half-values of the difference between the current densities of the anodic and cathodic scans with respect to the CV scan rate (10–90 mV s^−1^). To evaluate the stability of the as-synthesized catalyst, chronoamperometry (I–t measurement) and CV were conducted via constant-operation tests and accelerated-degradation tests, respectively. In the chronoamperometry, the changes of the cathodic current density values under a constant overpotential to achieve a current density of 10 mA cm^−2^ were recorded for 10 h. In the CV, the potential was applied from 0.0 to −0.45 V vs. RHE for 1000 cycles with a scan rate of 50 mV s^−1^.

## 3. Results

The MoS_2_ was synthesized on a Si substrate via a two-step growth process. First, a Mo film was deposited using an RF magnetron sputtering system (Figure 1a). Then, the Mo film was placed into a PECVD chamber for synthesizing MoS_2_ (Figure 1b). H_2_S gas accompanied by Ar was injected into the chamber as an S precursor. The thermal profile, including the times of plasma operation and H_2_S injection, is shown in Figure 1c.

The energetic conditions on the substrate (with regard to energy impinging on the substrate) can be affected by various plasma species, such as excited neutrals, radicals, ions, and electrons. These activated species set up and undergo different elementary reactions, e.g., chemical reactions, excitation, radiation, dissociation, recombination, and ionization. Thus, in plasma-based deposition processes, the effects of energy deposition for each incoming species on the substrate and the flux are crucial [26]. We used OES at the substrate to qualitatively investigate the emissions from the excited species in the plasma. Figure 1d,e shows the emission spectra measured under the operating conditions of H_2_S = 0 and 60 sccm, respectively. Figure 1d shows the intensities of various excited species of Ar (Ar I lines). The spectra were acquired at a distance of 15 cm from the ICP antenna. This position corresponded to the substrate position. With the introduction of H_2_S = 60 sccm (Figure 1e), some of the excited emission lines were suppressed owing to the emergence of other active species, such as S_2_ molecules, H_2_ molecules, atomic H (H_α_), and atomic S (S I 923.46 nm line), caused by the electron impact dissociation of molecular gases [27]. These active molecular species and atomic H and S significantly affected the microstructure of the film [26].

**Figure 2:**
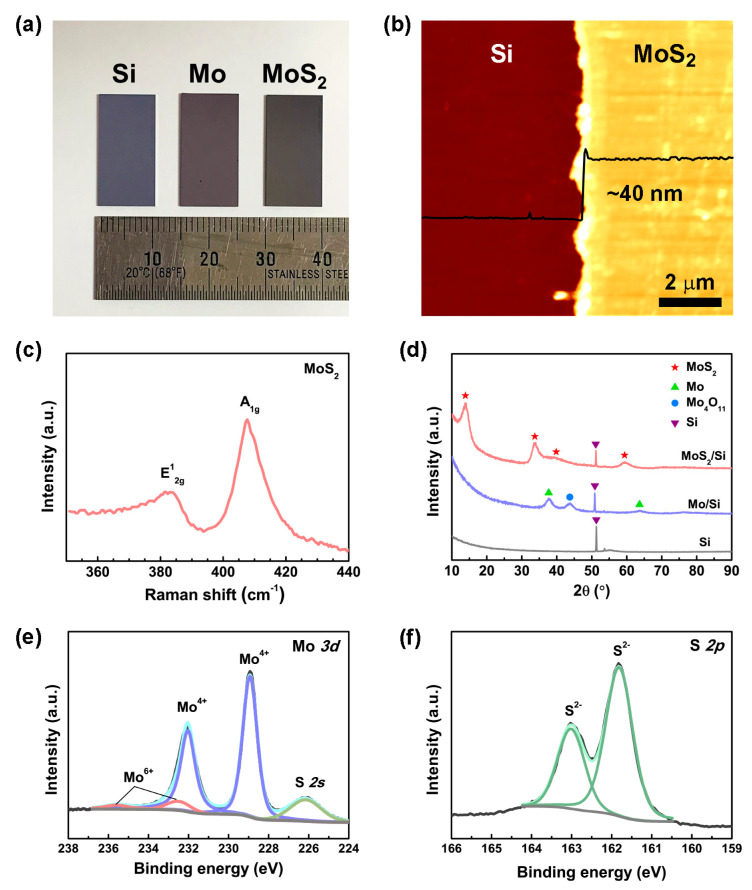
(**a**) Optical image of the Si substrate and Mo and MoS_2_ films. (**b**) Contact-mode AFM image of MoS_2_ and the relative height profile. (**c**) Raman spectra of the synthesized MoS_2_ film on the Si substrate, with two peaks at 382.9 and 407.6 cm^−1^, corresponding to the E^1^_2g_ and A_1g_ vibration modes, respectively. (**d**) XRD patterns of the bare Si, Mo film, and as-grown MoS_2_. XPS results for the synthesized MoS_2_ film in the binding energy intervals of (**e**) Mo *3d* and (**f**) S *2p*.

Figure 2a shows an optical image of the bare Si substrate, deposited Mo film, and grown MoS_2_, which had colors of light gray, purple, and dark gray, respectively. We measured the thicknesses of the Mo and MoS_2_ films using contact-mode AFM. The Mo film had a thickness of ~35 nm (Figure S2a). For the MoS_2_, the thickness was increased to ~40 nm because of the sulfurization process (Figure S2b). The crystallinity of the synthesized MoS_2_ was investigated using Raman spectroscopy. In Figure 2c, the Raman spectrum exhibits two typical peaks at 382.8 and 407.7 cm^−1^, corresponding to the in-plane (E^1^_2g_) and out-of-plane (A_1g_) vibration modes, respectively [28]. The frequency difference (∆*k*) between the two modes was approximately 24.9 cm^−1^, indicating bulk MoS_2_. XRD was utilized to study the phase composition of the deposited Mo film and grown MoS_2_ (Figure 2d). For the Mo film deposited on the Si substrate, diffraction peaks corresponding to Mo (JCPDS Card No. 1-1207) were detected, along with a weak diffraction peak corresponding to Mo_4_O_11_ (JCPDS Card No. 73-1538). The Mo_4_O_11_ may have been formed by oxidation during the Mo sputtering process. After sulfurization in PECVD, the diffraction peaks corresponded to MoS_2_ (JCPDS Card No. 37-1492), indicating that 2H-phase MoS_2_ was synthesized. The peak at 51.1° corresponded to the Si substrate.

The chemical bonding states and elemental composition of the grown MoS_2_ were measured using XPS. In Figure 2e, the core-level Mo *3d* spectrum exhibits strong doublets at 228.9 and 232.0 eV, corresponding to Mo^4+^ *3d*_5/2_ and Mo^4+^ *3d*_3/2_ (Mo–S bonding), respectively, and weak doublets at 232.5 and 235.6 eV, corresponding to Mo^6+^ *3d*_5/2_ and Mo^6+^ *3d*_3/2_ (Mo–O bonding), respectively [29]. The Mo^6+^ *3d* peaks indicate the presence of MoO_3_, which may have been formed by the exposure of MoS_2_ to air [30,31]. Calculations indicated that the atomic fraction of Mo^6+^ *3d* in the grown MoS_2_ was approximately 8.30%. Additionally, the core-level S *2p* spectrum exhibited doublets at 161.8 and 163.0 eV, corresponding to S^2−^ *2p*_3/2_ and S^2−^ *2p*_1/2_, respectively. In previous studies, 1T-phase MoS_2_ exhibited Mo *3d* and S *2p* doublets at a position 0.8–1.0 eV lower than those of 2H-phase MoS_2_ [32]. However, in the present study, the peak appearing at 228.9 eV was symmetric, and no additional peak was observed around 228 eV, indicating no 1-T phase component. The ratio of S^2−^ *2p* to Mo^4+^ *3d* was approximately 1.96, which closely matches the composition of MoS_2_.

**Figure 3:**
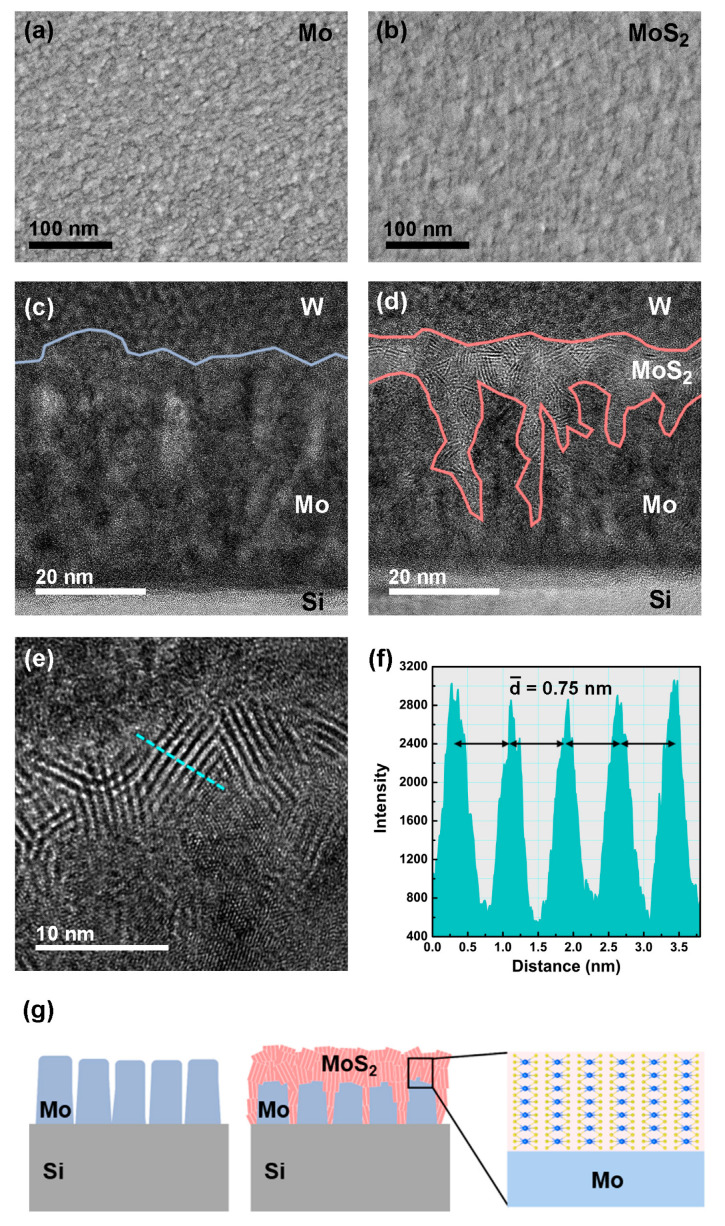
Typical SEM images of the (**a**) Mo film surface deposited via RF sputtering and (**b**) MoS_2_ film surface after the PECVD synthesis process. Cross-sectional TEM images of (**c**) Mo deposited on the Si substrate and (**d**) MoS_2_ grown on the Si substrate. (**e**) Cross-sectional high-resolution transmission electron microscopy (HRTEM) image of MoS_2_ with a layered structure. (**f**) Intensity profiles corresponding to the blue dotted line in (**e**). (**g**) Schematics of Mo deposited on the Si substrate and sulfurized MoS_2_.

The surface morphologies of the Mo and MoS_2_ films were examined using SEM (Figure 3a,b). The SEM images indicated that the synthesized MoS_2_ had larger grains and a more uniform surface than the Mo film. The RMS roughness values of the surfaces of the Mo and MoS_2_ films were also increased from 0.8 to 2.8 nm. (Figure S2c,d). Cross-sectional TEM images of the Mo and MoS_2_ films are presented in Figure 3c,d. In Figure 3c, the cross-section of the Mo film exhibits a pillar shape perpendicular to the Si substrate. After the sulfurization, the MoS_2_ grew along the top and side-edge surfaces of the Mo pillars, with a polycrystalline structure (Figure 3d). The elemental line profile obtained via cross-sectional HRTEM is shown in Figure S3.

The thickness of the MoS_2_ growing on the top surface of the Mo film was approximately 6–9 nm. In most previous studies, the thickness of MoS_2_ layers grown by sulfurizing Mo films could not exceed ~7 nm [33,34]. This limitation was possibly due to the diffusion ability of the S precursor. However, in the present study, the maximum depth of the MoS_2_ growing along the edge of the Mo pillars was ~35 nm, confirming that the Mo growing on the Si substrate had a pillar shape and that the Mo surface was converted into MoS_2_ by the sulfurization process. Schematics of the polycrystalline MoS_2_ growing on the Mo film are presented in Figure 3g. Many studies have indicated that increasing the number of active sites can enhance the efficiency of the HER [12,17]. Both theoretical and experimental results have suggested that the exposed edge sites of MoS_2_ exhibit higher HER activity than the basal planes [19]. Thus, the multiorientated MoS_2_ films grown in this study were expected to exhibit good HER performance.

As shown in the HRTEM image in Figure 3e, the polycrystalline MoS_2_ consisted of several layers, with an average interlayer spacing of ~0.75 nm (Figure 3f). The theoretical interlayer spacing of MoS_2_ is 0.615 nm, which is smaller than the value measured in this study [35]. It has been reported that increasing the interlayer spacing of MoS_2_ can reduce the Gibbs free energy of H adsorption and enhance the intralayer conductivity, which is beneficial for catalyzing the HER [36]. The reason for the large interlayer spacing of MoS_2_ in this study may be the PECVD growth method. The ICP-assisted MoS_2_ growth not only enhanced the reaction rate of H_2_S but also created defects in the MoS_2_ [13,25]. Additionally, the low growth temperature may be another reason for the large interlayer spacing. However, the exact reasons are unknown and require further study.

**Figure 4:**
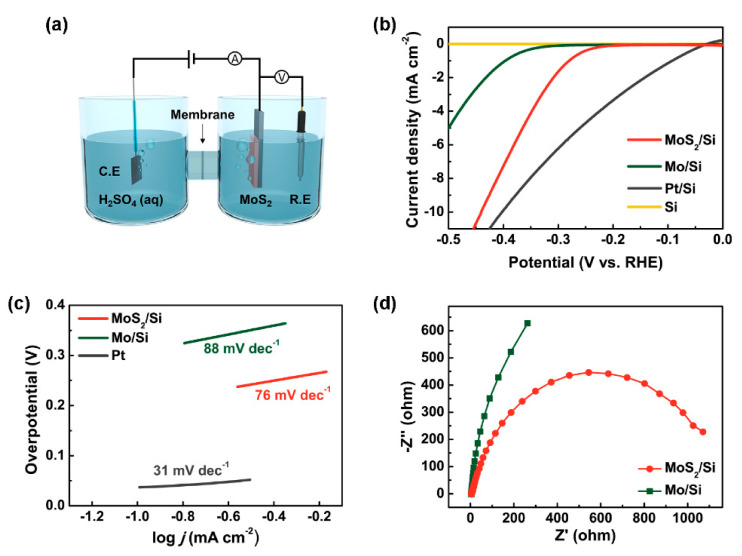
(**a**) Schematic of the hydrogen evolution reaction (HER) system. (**b**) Polarization curves of Si, Pt, Mo, and MoS_2_ in the 0.5 M H_2_SO_4_ electrolyte. The scan rate was 5 mV s^−1^, and the scan region was 0 to −0.5 V vs. reversible hydrogen electrode (RHE). (**c**) Corresponding Tafel plots obtained from the polarization curves. (**d**) Nyquist plots for Mo and MoS_2_; the applied potential was −0.2 V vs. RHE.

Electrochemical measurements were performed using a standard three-electrode system, as shown in Figure 4a. A proton-exchange membrane was inserted between the counter electrode and the working electrode to separate them. This is because the dissolution of Pt on the counter electrode and the regeneration via the formation of Pt nanoparticles on the working electrode may have led to inaccurate results for the HER performance. It was previously reported that the HER performance of Pt nanoparticles on MoS_2_ nanosheets was enhanced by activating the inert basal plane of MoS_2_ [37]. Thus, it is recommended to adopt a proton-exchange membrane or replace the counter electrode with other materials such as glassy C. In this study, the proton-exchange membrane was used to preserve the high purity of the counter electrode.

Cathodic polarization curves are shown in Figure 4b. In the case of Si, the HER performance was poor. After the deposition of Mo on Si, better performance was observed. After sulfurization via PECVD, the Mo-deposited Si was converted into MoS_2_, which was beneficial for the HER. The overpotential of a catalyst is generally selected as the value achieving a current density of 10 mA cm^−2^, which corresponds to an efficiency of 12.3% for a solar-to-H device in solar water splitting [38]. Additionally, this criterion has been widely accepted for comparing the catalytic performance in previous studies. The overpotential of the MoS_2_/Mo/Si sample was 0.45 V, which was slightly higher than that of Pt (0.4 V).

To identify the rate-determining step of the HER process, the Tafel plot was obtained by replotting Figure 4b (potential vs. logarithm of current density), as shown in Figure 4c. In acidic solutions, there are three principal reactions: the first step is the adsorption of protons on active sites of the catalyst (Volmer reaction), and the next step involves either the desorption of protons on the catalyst after the combination of nearby protons in the solution (Heyrovsky reaction) or the binding of two protons on the catalyst (Tafel reaction) [39]. It is well known that the Tafel slope is 120, 40, and 30 mV dec^−1^ when the rate-determining step is the Volmer reaction, Heyrovsky reaction, and Tafel reaction, respectively. Therefore, the Tafel slope is important for probing the mechanism. Mo/Si, MoS_2_/Mo/Si, and Pt exhibited Tafel slopes of 88, 76, and 31 mV dec^−1^, respectively. The Tafel slope of Pt in this study indicated that the rate-determining step of Pt was the Tafel reaction, and it was similar to previously reported values [10]. Mo/Si and MoS_2_/Mo/Si had the same rate-determining step, the Heyrovsky reaction. However, the Tafel slope of MoS_2_/Mo/Si was lower than that of Mo/Si, indicating faster H evolution due to higher overpotential of MoS_2_/Mo/Si [40]. Several approaches have been used for synthesizing MoS_2_, including CVD [15,16,17,18,41,42], the hydrothermal method [21,22,43], and ALD [20]. The overpotential and Tafel slope of MoS_2_/Mo/Si in this study were comparable to the previous reports [15,17,22,44,45].

To evaluate the interfacial characteristics of the catalyst, EIS was performed, and the Nyquist plots of Mo/Si and MoS_2_/Mo/Si are shown in Figure 4d. The Nyquist plots of all the catalysts were obtained at an OCP in the frequency region of 100 kHz to 100 MHz, with an excitation signal of 10 mV (RMS). The point in the Nyquist plots in the vicinity of the origin indicates the series resistance (*R*_s_), and the diameter of the semicircle indicates the charge-transfer resistance (*R*_ct_) [46]. The *R*_s_ is related to the electrical-transport property of a catalyst, and the *R*_ct_ is associated with the catalytic kinetics at the interface between the catalyst and the electrolyte [47]. MoS_2_/Mo/Si had a small *R*_ct_, indicating enhanced electron-transfer kinetics, whereas its *R*_s_ was similar to that of Mo/Si. Therefore, MoS_2_/Mo/Si had better charge transport from the Si substrate to its catalytic sites.

Although current density values normalized by the total geometric surface area are used to compare the performance of different catalysts, they do not indicate which catalyst has the highest intrinsic activity. To understand the intrinsic catalytic performance of a catalyst, the number of active sites must be determined. Mass loading, determination of the total surface area via Brunauer–Emmett–Teller (BET) theory, and measurement of the ECSA via estimation of the double-layer capacitance (*C_dl_*) are commonly used to determine the number of active sites of a catalyst. However, it is well known that MoS_2_ in the 2H-phase has activity only on edge sites, not on the basal plane [48]. Therefore, normalizing the total electrode activity by the catalyst mass may lead to an underestimation of the intrinsic activity of the MoS_2_ catalyst. Additionally, it is not critical to consider the MoS_2_ mass loading or the cost of the catalyst during the device fabrication, because MoS_2_ is not a precious material such as Pt. Measuring the total catalytic surface area rather than the mass is a better strategy because the number of active sites often increases with the catalyst surface area. Two approaches are commonly used to measure the surface area of nonprecious catalysts. One option is gaseous adsorption techniques such as the BET method, and the other is electrochemical measurements. The former is appropriate for porous materials with large surface areas [49], but it may overestimate the surface area by including the electrically insulating region where the catalytic reaction cannot occur [50]. The latter measures the ECSA directly. The ECSA is generally determined by measuring the non-Faradaic current associated with electrochemical double-layer charging at the catalyst surface, and the capacitive current at the middle value in the potential region (difference between the currents of the cathodic and anodic scans) is calculated and compared with reference flat materials, for estimating the roughness factor [51].

**Figure 5:**
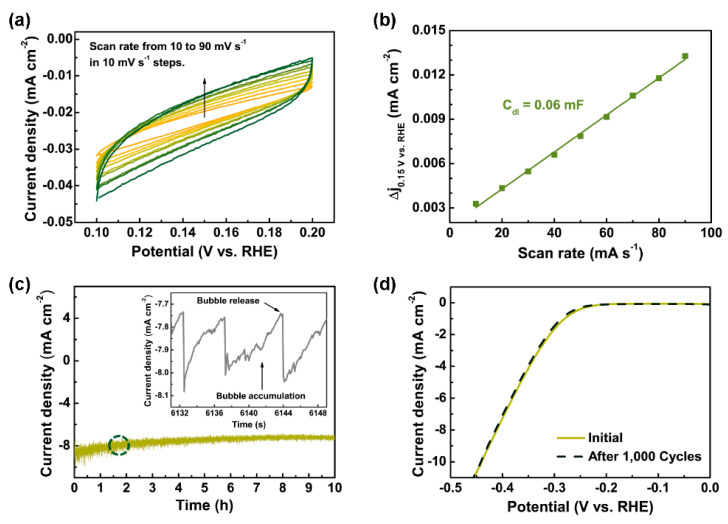
(**a**) CV response of MoS_2_ tested at various scan rates ranging from 10 to 90 mV s^−1^ in the potential range of 0.1–0.2 V. (**b**) Capacitive current at 0.15 V with respect to the scan rate for the MoS_2_ film in the 0.5 M H_2_SO_4_ electrolyte. (**c**) Time dependence of the current density for 10 h, under the static overpotential corresponding to a current density of 10 mA cm^−2^ based on the recorded value in the polarization curve. After 10 h, the current density was 83% of the initial value. Inset: magnified image of the area indicated by the dashed circle. (**d**) Cycling stability test results, indicating negligible current loss after 1000 cycles. The light and dark green lines are the polarization curves of the sample before and after cycling, respectively.

As shown in Figure 5a, CV was performed in the potential region of 0.10–0.20 V vs. RHE, where the non-Faradaic process occurred [52]. The current density values of MoS_2_/Mo/Si, which indicated the double-layer capacitance at the surface of MoS_2_, were measured at various scan rates. The difference in the current density values between the cathodic and anodic scans increased as the scan rate increased. To evaluate the double-layer capacitance, the half-value of the current density at 0.15 V vs. RHE was plotted with respect to the scan rate, as shown in Figure 5b. The results revealed that the double-layer capacitance of MoS_2_ was 0.06 mF cm^−2^, which is consistent with the previously reported value for MoS_2_ with a flat morphology [51].

To ensure the long-term stability of MoS_2_ in this study, a constant-operation test was performed via chronoamperometry. The time evolution of the current density values of MoS_2_ was recorded for 10 h, under a constant potential. The results are shown in Figure 5c and indicated that MoS_2_ still exhibited good performance after 10 h. The inset shows a magnified plot of the selected region enclosed by a dotted line, clearly indicating the change of the current density value during the bubble accumulation and release cycle. Along with chronoamperometry, an accelerated-degradation test was performed via CV (Figure 5d). In each cycle, a potential in the range of 0.0 to −0.45 V vs. RHE was applied with a scan rate of 50 mV s^−1^, and this was repeated 1000 times. The polarization curves before and after the cyclic test were compared. The two curves were nearly identical, indicating that the MoS_2_ had good durability.

## 4. Conclusions

Multiorientated polycrystalline MoS_2_ films were synthesized for HER applications. The two-step process consisted of RF sputtering of Mo and PECVD sulfurization. Using the ICP-assisted PECVD process, the growth temperature was reduced to 500 °C. Furthermore, the composition of gaseous species during the sulfurization was monitored using OES, making the synthesis process of MoS_2_ more controllable. The grown MoS_2_ was multiorientated, with a large interlayer spacing of ~0.75 nm, which provided abundant active sites, reduced the Gibbs free energy of H adsorption, and enhanced the intralayer conductivity. The catalytic HER performance was evaluated, revealing an overpotential value of 0.45 V for a current density of 10 mA cm^−2^ and a Tafel slope of 76 mV dec^−1^, which are comparable to previous reports. Our two-step process is scalable, and the grown MoS_2_ films are expected to be applied in industry.

## Supplementary Materials

The following are available online at https://www.mdpi.com/2079-4991/10/8/1465/s1, Figure S1. Schematic of the RF magnetron sputtering system, Figure S2. Thicknesses and morphologies of the Mo film and MoS_2_ grown on the Si substrate, Figure S3. Cross-sectional HRTEM image of MoS_2_ synthesized on the Si substrate and elemental line profile of MoS_2_ corresponding to the white line.
