# Supplementary material for: Growth of Multiorientated Polycrystalline MoS2 Using Plasma-Enhanced Chemical Vapor Deposition for Efficient Hydrogen Evolution Reactions
Source: Nanomaterials (Basel). 2020 Jul 27;10(8):1465. doi: 10.3390/nano10081465 (PMC7466528; doi:10.3390/nano10081465)
Supplement: Supplementary file 1 [file nanomaterials-10-01465-s001.pdf]

nanomaterials

MDPI

Supplementary Materials

Growth of Multiorientated Polycrystalline MoS_2 Using Plasma-Enhanced Chemical Vapor Deposition for Efficient Hydrogen Evolution Reactions

Na Liu [1,†], Jeonghun Kim [1,†], Jeonghyeon Oh [1], Quang Trung Nguyen [1], Bibhuti Bhusan Sahu [2], Jeong Geon Han [3] and Sunkook Kim [1,*]

[1] School of Advanced Materials Science & Engineering, Sungkyunkwan University (SKKU), Suwon, Gyeonggi-do 16419, Korea; naliufit9@gmail.com (N.L.); jhuni7803@gmail.com (J.K.); raymond.lucid@gmail.com (J.O.); nqtrungdt@gmail.com (Q.T.N.)

[2] Graduate School of Engineering, Center for Low-Temperature Plasma Sciences, Department of Electrical Engineering and Computer Science, Nagoya University, Nagoya 464-8601, Japan; bibhutisahu@gmail.com

[3] Thai-Korea Research Collaboration Research Center (TKRCC), Science and Technology Park (STeP), Chiang Mai University, Chiang Mai 50200, Thailand; hanjg5445@gmail.com

* Correspondence: seonkuk@skku.edu

† These authors contributed equally to this work.

Figure S1 presents a schematic of the experimental system, indicating the crucial components used in the present study for the deposition of the ultrathin Mo film. The system comprised a cylindrical plasma chamber, vacuum system, circular and planar (4 inches) magnetron, gas-flow arrangement, substrate heating system, and load lock chamber. A combination of a turbo molecular pump and a rotary pump evacuated the chamber to a base vacuum of ~1×10^{-6} Torr. The load lock chamber was used to load and unload the samples without breaking the vacuum condition before and after the deposition, respectively.

The magnetron was equipped with the Mo target, with a water-cooling arrangement. We used Ar as the experimental gas at a constant applied RF power of 160 W (this value corresponds to a power density of 2 W cm^{-2}) for the plasma generation and the film deposition. We conducted presputtering of the target for 5 min, closing the shutter prior to every deposition. The distance from the center of the target to the center of the substrate table was 14 cm. The substrate position was adjusted using a motorized controller. The operating pressure for the deposition was 7 mTorr. Each substrate was ultrasonically cleaned before the deposition. The film thickness was ~35 nm for the transfer process for the sulfurization via ICP-assisted PECVD.

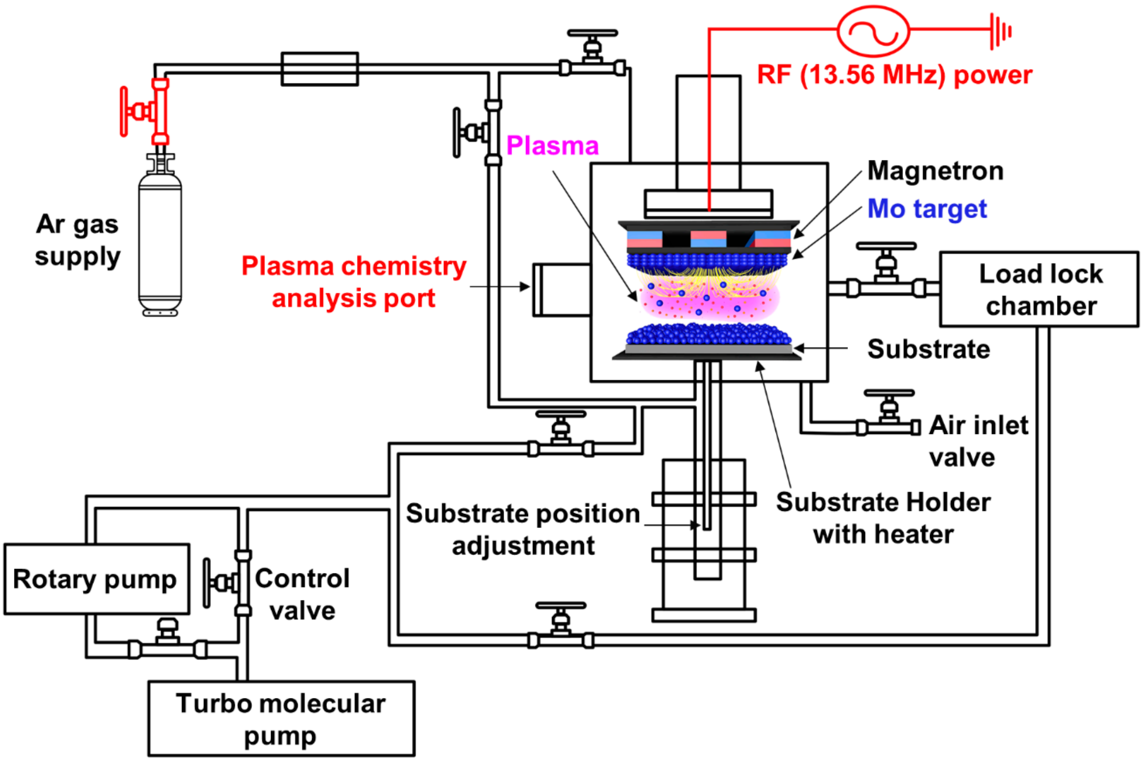

Figure S1. Schematic of the RF magnetron sputtering system.

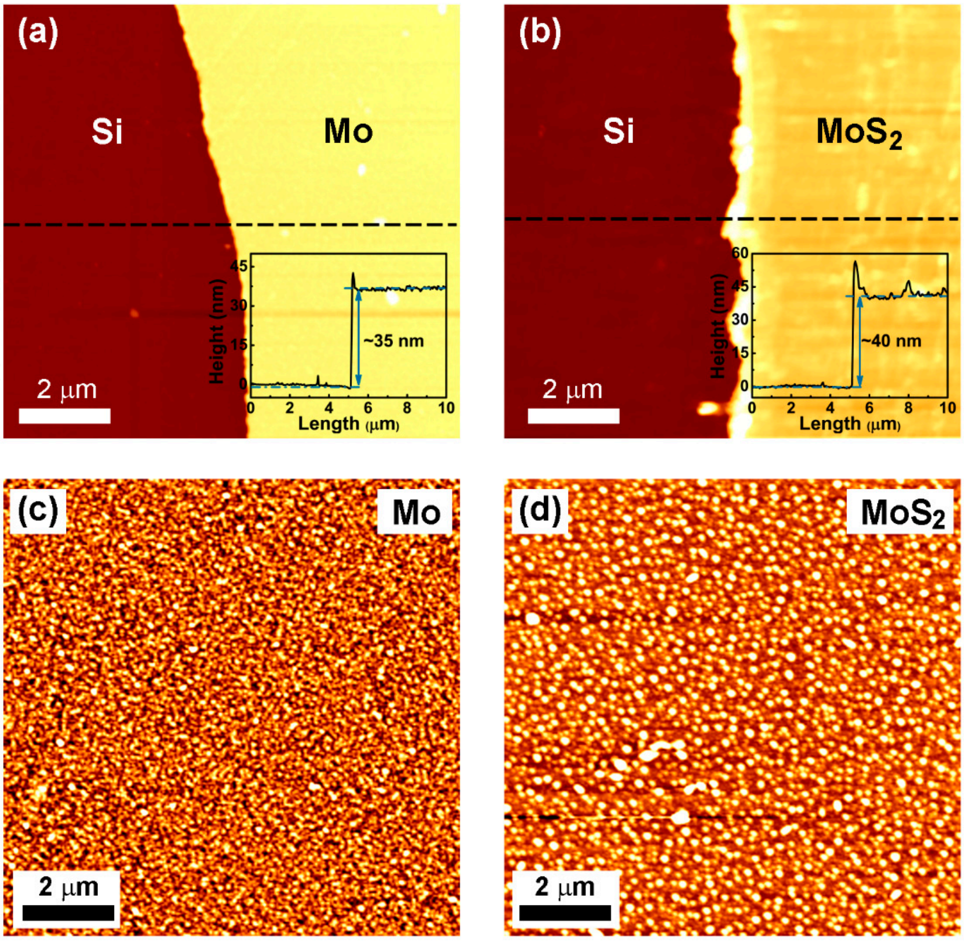

Figure S2. Thicknesses and morphologies of the (**a**,**c**) Mo film and (**b**,**d**) MoS_2 grown on the Si substrate.

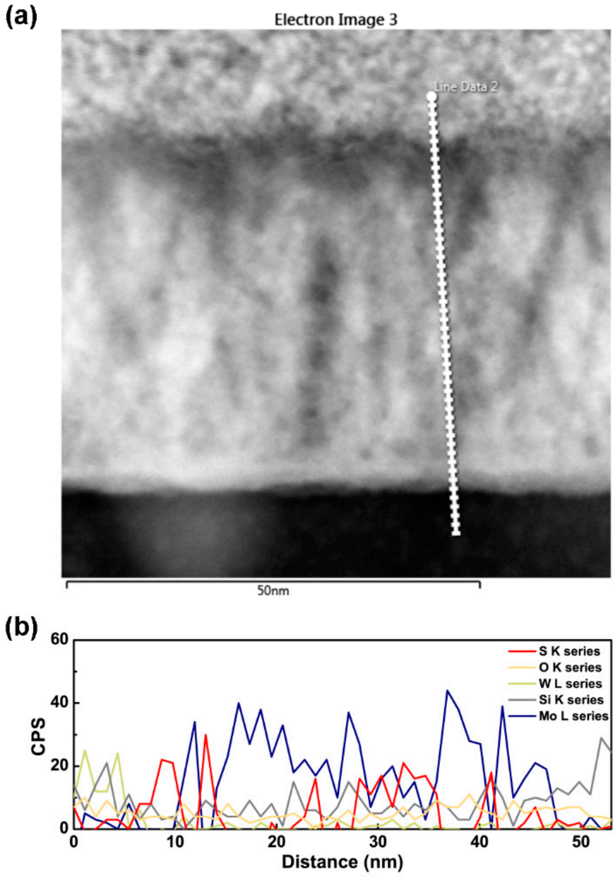

Figure S3. (**a**) Cross-sectional HRTEM image of MoS_2 synthesized on the Si substrate. (**b**) Elemental line profile of MoS_2 corresponding to the white line in (**a**).

© 2020 by the authors. Licensee MDPI, Basel, Switzerland. This article is an open access article distributed under the terms and conditions of the Creative Commons Attribution (CC BY) license (http://creativecommons.org/licenses/by/4.0/).
